# Range‐wide genomic data synthesis reveals transatlantic vicariance and secondary contact in Atlantic cod

**DOI:** 10.1002/ece3.4672

**Published:** 2018-11-16

**Authors:** Robert Fairweather, Ian R. Bradbury, Sarah J. Helyar, Mark de Bruyn, Nina O. Therkildsen, Paul Bentzen, Jakob Hemmer‐Hansen, Gary R. Carvalho

**Affiliations:** ^1^ Department of Biology Dalhousie University Halifax Nova Scotia Canada; ^2^ School of Biological Sciences Bangor University Bangor UK; ^3^ Science Branch, Department of Fisheries St John’s, Newfoundland and Labrador Canada; ^4^ Institute of Global Food Security Queen’s University Belfast Belfast UK; ^5^ School of Life and Environmental Sciences The University of Sydney Sydney New South Wales Australia; ^6^ Department of Natural Resources Cornell University Ithaca New York; ^7^ Section for Marine Living Resources, National Institute for Aquatic Resources Technical University of Denmark Silkeborg Denmark

**Keywords:** Atlantic cod, fish, *Gadus morhua*, marine, phylogeography, synthesis

## Abstract

Recent advances in genetic and genomic analysis have greatly improved our understanding of spatial population structure in marine species. However, studies addressing phylogeographic patterns at oceanic spatial scales remain rare. In Atlantic cod (*Gadus morhua*), existing range‐wide examinations suggest significant transatlantic divergence, although the fine‐scale contemporary distribution of populations and potential for secondary contact are largely unresolved. Here, we explore transatlantic phylogeography in Atlantic cod using a data‐synthesis approach, integrating multiple genome‐wide single‐nucleotide polymorphism (SNP) datasets representative of different regions to create a single range‐wide dataset containing 1,494 individuals from 54 locations and genotyped at 796 common loci. Our analysis highlights significant transatlantic divergence and supports the hypothesis of westward post‐glacial colonization of Greenland from the East Atlantic. Accordingly, our analysis suggests the presence of transatlantic secondary contact off eastern North America and supports existing perspectives on the phylogeographic history of Atlantic cod with an unprecedented combination of genetic and geographic resolution. Moreover, we demonstrate the utility of integrating distinct SNP databases of high comparability.

## 1 INTRODUCTION

The contemporary distribution of marine species has been shaped by both current and historical patterns in ocean climate and habitat availability (Kettle, Morales‐Muñiz, Roselló‐Izquierdo, Heinrich, & Vøllestad, 2011; Perry, Low, Ellis, & Reynolds, 2005). Genetic divergence across ocean basins has been reported in many marine species consistent with the isolation of populations during the Pleistocene (Hewitt, 2003; Maggs et al., 2008), when periods of climatic cooling and glacial advance acted to segregate marine populations into glacial refugia (Provan & Bennett, 2008). For example, the last glacial maximum (LGM,~21 kya) saw the loss of most subarctic shelf sea habitat in the North Atlantic due to advancing glacier and lower sea levels (Lambeck, Esat, & Potter, 2002; Mitrovica, 2003; Peltier, 1994). Many shelf sea species persisted in glacial refugia mostly at more southerly latitudes in the East and West Atlantic, before expanding north during glacial retreat (Hewitt, 2003; Maggs et al., 2008; Provan & Bennett, 2008). Signatures of glacial isolation, subsequent range expansion, and secondary contact have been observed in a variety of Atlantic taxa including fish (Chevolot, Hoarau, Rijnsdorp, Stam, & Olsen, 2006; Souche et al., 2015), benthic invertebrates (Jolly, Viard, Gentil, Thiébaut, & Jollivet, 2006; Young, Torres, Mack, & Cunningham, 2002), and macroalgae (Hoarau, Coyer, Veldsink, Stam, & Olsen, 2007; Provan, Wattier, & Maggs, 2005). As a result of human‐induced climate change, contemporary distributions of marine species are anticipated to shift dramatically once again, with profound ecological and economic consequences (Stanley et al., 2018; Wisz et al., 2015). The analysis of climatically induced changes in past distributions can provide valuable insights into predicted future responses (Beaugrand, Edwards, Raybaud, Goberville, & Kirby, 2015).

Phylogenetic analysis of contemporary populations has become a valuable tool in assessing changes in distributions in marine species (Maggs et al., 2008). However, for marine fishes, large‐scale range‐wide assessments of population structure and post‐glacial colonization are often challenging due to the logistical difficulties of sampling marine species at ocean basin scales and uncertain comparability of length‐based genetic markers (Lahood, Moran, Olsen, Grant, & Park, 2002). The last decade has seen a shift in the approaches employed from microsatellites and mitochondrial DNA to panels of single‐nucleotide polymorphisms (SNPs). In addition to their increasingly low cost and genome‐wide information content (Fuentes‐Pardo & Ruzzante, 2017; Helyar et al., 2011), the simple format of essentially binary SNP data also facilitates the posthoc integration of independently collected genotype datasets (Helyar et al., 2011; Morin, Luikart, & Wayne, 2004). Genetic data synthesis has already been used in human phylogeography to supplement existing datasets with genotypes from previously unrepresented regions (Jinam et al., 2012). Similarly, in marine fish conservation, the ease of SNP synthesis has been used to encourage the use of universal genetic databases for taxa with transboundary ranges, such as tunas (Albaina et al., 2013) and Atlantic salmon (Jeffery et al., 2018). The increasing number of population genetic studies for many species and the prospective ease with which combined datasets may be generated presents an opportunity for collaborative approaches to address range‐wide phylogeographic questions.

Atlantic cod (*Gadus morhua*) is a benthopelagic gadid found throughout the shelf seas of the North Atlantic, ranging from the Norwegian Arctic to as far south as the Bay of Biscay in the East Atlantic and North Carolina in the west (Robichaud & Rose, 2004; Rose, 2007). Ancestry of contemporary populations can be traced to the pre‐Wisconsin interglacial period 200–250 kya (Lait, Marshall, & Carr, 2018). During the LGM, cod were separated into East and West Atlantic glacial refugia before advancing northward during glacial retreat, with recolonization of Greenland hypothesized to have resulted from westward dispersal from the East Atlantic (Bigg et al., 2008). Previous analyses of range‐wide population structure in cod undertaken using mtDNA, allozymes, microsatellites, and SNPs suggested pronounced genetic differentiation between contemporary East and West Atlantic populations (Berg et al., 2017; Bradbury et al, 2010; Bradbury et al., 2013; Carr & Marshall, 2008; Lait et al., 2018; Mork, Ryman, Ståhl, Utter, & Sundnes, 1985;). However, earlier studies have been limited by genomic and geographic coverage (Mork et al., 1985; O'Leary, Coughlan, Dillane, McCarthy, & Cross, 2007; Pogson, Mesa, & Boutilier, 1995), while others tend to lean heavily on either one or the other side of the Atlantic (Berg et al., 2017; Carr & Marshall, 2008; Lait et al., 2018).

Here, we address these limitations by using a genetic data‐synthesis approach to investigate range‐wide contemporary population structure in Atlantic cod. In our study, we address the difficulties of gathering representative samples from the entire species distribution through a synthesis of data from several independent regional studies. Using these data, we examine phylogeographic history in cod throughout the Atlantic at unprecedented geographic resolution. In particular, we aim to (a) re‐examine the evidence of a genetic break between the East and West Atlantic, (b) verify the evidence for recolonization of Greenland from the East Atlantic, and (c) investigate secondary contact between North American and European cod. Additionally, our analysis provides a case study of the utility of a genomic data‐synthesis approach for future ecological studies.

## 2 METHODS

### 2.1 Data synthesis

**Figure 1:**
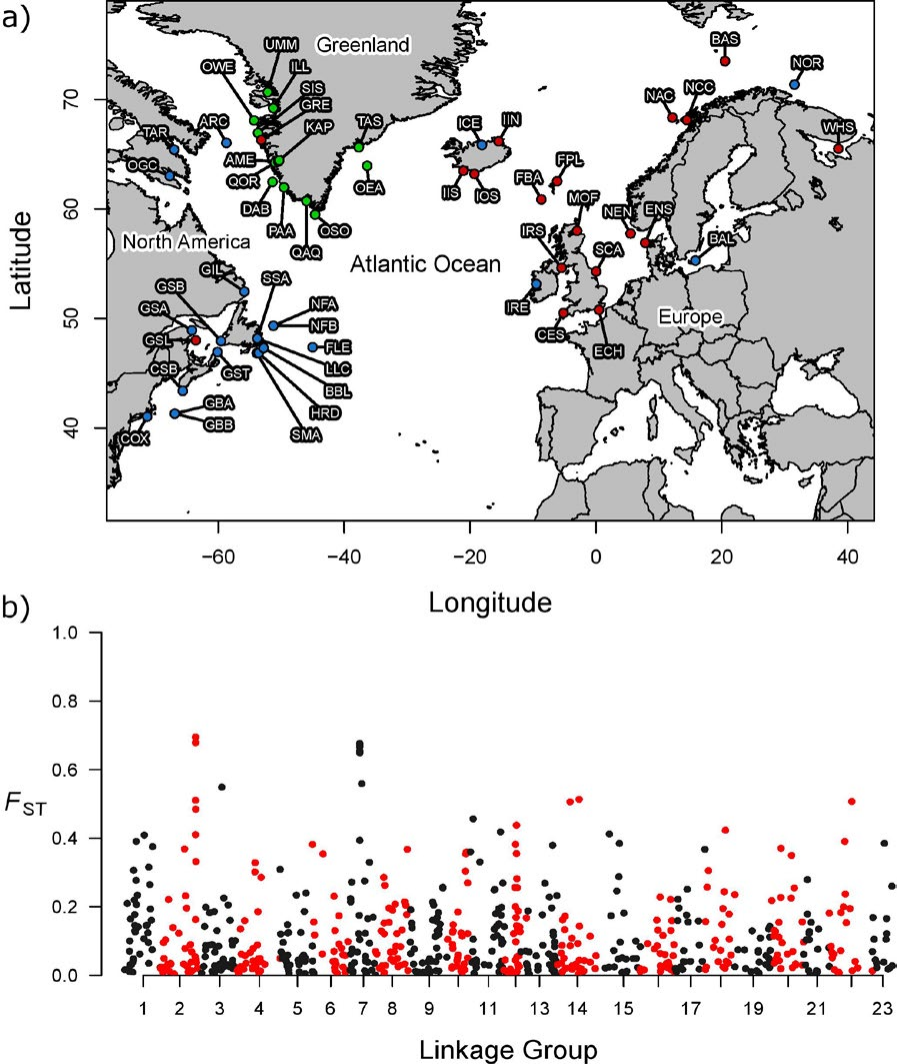
An overview of the data presented in this study. (a) Geographic distribution of samples color coded by dataset of origin, RED = FishPopTrace (FPT), BLUE = Cod Genomics Broodstock Project (CGP), GREEN = Therkildsen et al. (2013). Sample attributes are in the Supporting information Appendix S1. (b) Genomic distribution of 796 SNP panel against per‐locus *F*_ST_. Alternating colors indicate transitions between linkage groups. Note that not all SNPs are mapped to a linkage group (further details in the Supporting information Appendix S1)

A dataset of 1,494 individuals from 54 sampling replicates was assembled from datasets provided by the FishPopTrace (FPT) Consortium (Nielsen et al., 2012), the Cod Genomics Broodstock Project (CGP) (Bradbury et al., 2013), and the authors of Therkildsen et al. (2013), and covering the East Atlantic, West Atlantic, and Greenland, respectively (Figure 1a). Individuals from all datasets had been genotyped for 972 shared SNPs originally identified in a Canadian broodstock (Hubert, Bussey, Higgins, Curtis, & Bowman, 2009). Sampling took place between 1996 and 2010 as part of scientific surveys and commercial harvests, and fish were typically in spawning condition, except for Arctic samples where fishing was restricted to summer months. All samples comprised blood or tissue from between 9 and 40 individuals per location. Further details of sampling and genotyping protocols can be found in Nielsen et al. (2012), Bradbury et al. (2013) and Therkildsen et al. (2013), and references therein.

Sequence strand designations between CGP and FPT components of the data were found to be inconsistent (a known issue with the Illumina Golden Gate platform used for Genotyping the FPT data) and, in the absence of genotyping records, ambiguous loci (A/T and C/G polymorphisms) were corrected manually by comparison of minor allele frequencies (MAF) between CGP and FPT samples from geographically proximate locations (IRE/IRS, GST/GSL, and NOR/BAS). We ground truthed this approach by comparing allele frequencies of unambiguous loci between datasets to determine the rate of matched minor alleles at cumulative 0.05 MAF intervals. The probability of incorrect assignment at each interval was calculated as the cumulative count of loci occurring between 0 and interval x with a CGP frequency >0.5, divided by the cumulative total up to that interval. Error rates were subtracted from 1 and averaged across all three locales to estimate confidence, and a traditional confidence interval of 0.95 was used to define the MAF threshold. Thus, where the frequency of a given allele was greater or less than the threshold at both locations, matched strands were assumed, while where allele frequency was less than the threshold in one dataset and greater in the other, mismatched strands were corrected. Final inclusion depended on a consistent consensus and an average confidence interval >0.95 at all three locations. Missing data were calculated per locus using genalex (Peakall & Smouse, 2012) and loci exhibiting high concentrations of missing data (≤60% of loci successfully genotyped in any sample) or missing significant amounts of data (≥15%) in more than five samples were discarded. Departure from Hardy–Weinberg equilibrium (HWE) was tested in all samples using the Monte Carlo Markov Chain procedure implemented in Pegas (Paradis, 2010) with 105 permutations. Multiple testing was corrected for using the R‐package qvalue (Storey & Tibshirani, 2003). SNPs that remained significantly divergent in more than three samples following FDR correction and these were discarded. To reduce bias attributable to inconsistencies between datasets, three pairs of samples representing geographic replicates between the FPT and CGP datasets were scanned for high per‐locus *F*_ST_ and the degree of common occurrence in the 5% upper *F*_ST_ quantiles was computed using venndiagram (Chen & Boutros, 2011). Loci occurring in the upper 5% in more than one replicate, with high occurrence of missing data, or deviating significantly from Hardy–Weinberg expectations were discarded. Due to more restricted spatial overlap between the Greenland dataset and other samples, this analysis could not be repeated with the Greenland data. However, as Greenland samples were genotyped in the same lab and according to the same protocol as FPT data and comprise a direct subset of the FPT markers as well as sharing some individuals, strong agreement was anticipated even in the absence of this step.

### 2.2 Population genetics

Expected heterozygosity and observed heterozygosity were calculated in all samples using GenAlEx. Pairwise *F*_ST_ was calculated between all samples via the Weir and Cockerham (1984) method implemented by hierfstat (Goudet, 2005). Isolation by distance (IBD) was evaluated using a Mantel test to test the strength of relationship between *F*_ST_/(1‐*F*_ST_) and shelf sea distance. To control for the possibility that IBD detected in the full dataset is a product of geographic distance between divergent regions, IBD was evaluated individually within the Europe, Greenland/Iceland, and North America. Mean *F*_ST_ was calculated within each region and compared between regions using a Welch *t* test. Population structure was further investigated by running structure 2.3.4 (Pritchard, Stephens, & Donnelly, 2000) with the admixture model. A burn‐in period of 100,000 followed by an MCMC run of 500,000 iterations was used. Values of *K* ranged from 1 to 6 with three runs per *K*‐value. Optimal *K* was identified by the change in likelihoods (Δ*K*, Evanno, Regnaut, & Goudet, 2005), and results were analyzed using clumpack (Kopelman, Mayzel, Jakobsson, Rosenberg, & Mayrose, 2015). structure was run in a hierarchal fashion, evaluating first all populations, and then East and West Atlantic separately. For East and West runs, we experimented with the inclusion and exclusion of samples admixed in the range‐wide run, including the Davis Strait, Flemish Cap, and Arctic Lakes. Discriminate Analysis of Principle Components (DAPC) (Jombart, Devillard, & Balloux, 2010) was implemented using the R‐package adegenet (Jombart, 2008). Clusters were identified by running successive *K*‐means clustering for *K* = 1:60, and the best supported number of clusters was identified through comparison of Bayesian Information Criteria (BIC). DAPC was then used to describe the relationship between the inferred clusters. To examine the effects of ambiguous loci retention, population structure results were compared between the full panel and unambiguous loci only.

To filter the effects of selection and focus on neutral evolution, *F*_ST_ outlier loci were filtered using two approaches. First, a Bayesian approach was implemented using the software bayescan v.2.1 (Foll & Gaggiotti, 2008) with default settings, 10,000 iterations, and a burn‐in period of 500,000. Initially, the program was run on the entire dataset, resulting in a very large number of outliers (nearly half the loci). In a subsequent run, we accounted for transatlantic population structure by analyzing European and North American samples separately. Individuals from the Flemish Cap were included with Europe as population structure analysis indicated European affiliation. Samples from Lake Tariujarusiq and Lake Ogac were excluded from bayescan analysis to avoid confounding our result with genetic drift. Second, a hierarchal island model was implemented using arlequin v.3.5.2.2 (Excoffier & Lischer, 2010). The default numbers of simulations and demes per group were used, and the number of groups was set to the maximum permitted (50) to approximate the 54 samples in the data. As arlequin permits a priori input regarding expected neutral structuring, eight groups were defined using the eight clusters approximated in the DAPC analysis.

### 2.3 Migration

To examine migration across the Atlantic, samples were grouped according to geographic proximity and their connectivity estimated through population structure analysis, although care was taken not to overaggregate samples. Direction and magnitude of migration based on *G*_ST_ and number of migrants (Alcala, Goudet, & Vuilleumier, 2014) were estimated using the divMigrate method (Sundqvist, Keenan, Zackrisson, Prodöhl, & Kleinhans, 2016) implemented in diversity (Keenan, McGinnity, Cross, Crozier, & Prodöhl, 2013) with 1,000 bootstrap replicates. Additionally, gene flow was estimated for the same groups using treemix (Pickrell & Pritchard, 2012) to model evolutionary history with 1,000 bootstrap replicates and up to four prospective migration events compared based on residuals.

## 3 RESULTS

### 3.1 Data synthesis

When combined, 2,392 unique loci were found across the three datasets, of which 972 were common to all. For these, 106 loci exhibiting high concentrations of missing data (≤60% of loci successfully genotyped in any sample) or missing significant amounts of data (≥15%) in more than five samples were discarded. Out of 42,984 tests for HWE, 1,259 proved significant (*p* ≤ 0.05), with the highest concentrations in samples from GSL, OWE, and QAQ (55, 46, and 39 loci, respectively). However, only 81 observations remained significant after correction for FDR (*q* ≤ 0.05) and these were mostly distributed evenly among samples. Only three SNPs remained significantly divergent in more than three samples following FDR correction, and these were discarded. Average* F_ST_* between the same geographic replicates was expectedly low (0.012, 0.012, and 0.009 for pairs representing the Gulf of Saint Lawrence, Irish Sea, and Barents Sea, respectively), indicating an overall homogeneity between datasets. Five loci that appeared in the upper 5% *F*_ST_ quantile in more than one of these locations were discarded. Comparison of allele frequencies in unambiguous loci at the same locations revealed that an MAF threshold of <0.45 retained an average confidence of 97.09% and >95% confidence at all three locations individually. Strand correction of ambiguous (A/T and C/G markers) based on this threshold allowed the retention of 163 loci.

### 3.2 Population genetics

**Figure 2:**
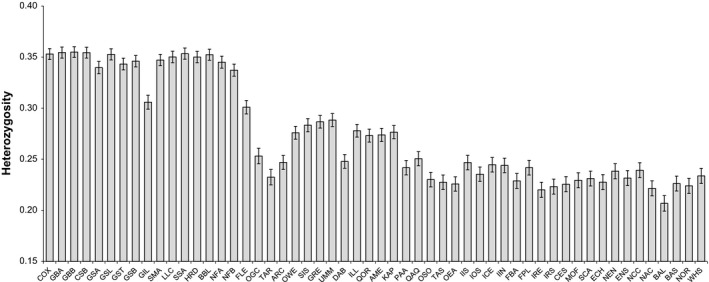
Observed heterozygosity per sample ±1 *SD*. Samples arranged approximately west to east

**Figure 3:**
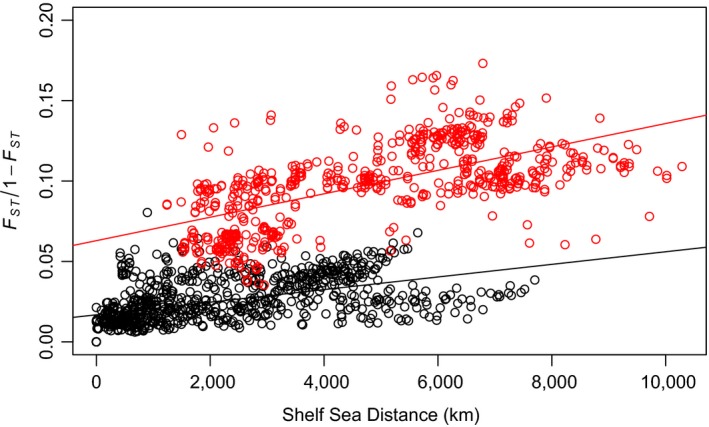
Pairwise FST/(1‐FST) plotted against shelf sea distance. Comparisons between the West and East Atlantic are in red. Comparisons within the West or East Atlantic are in black

Population genetic analysis commenced with 796 genome‐wide markers (Figure 1b). Heterozygosity within samples ranged from 0.355 on Georges Bank to 0.207 in the semi‐isolated Baltic Sea and was also especially low in Lake Ogac and Lake Tariujarusiq (0.253 and 0.232, respectively). A decline in heterozygosity was observed from North America to Europe, with a particularly marked reduction in the transition between Atlantic Canada and Europe (Figure 2). Heterozygosity for unambiguous loci only was tightly correlated with observations for the full panel (*r* ^2^ = 0.9986, *p* < 0.001) and was consistently higher for each sample, although this difference was not significant (Welch *t* test; *t *= −1.094, *df *= 105.78, *p* = 0.277). Pairwise comparisons of *F*_ST_ with and without ambiguous loci were also tightly correlated (*r* ^2 ^= 0.9982, *p* < 0.001). Differences were always smaller than 0.006 and were less than 0.001 in 66% of cases. Pairwise *F*_ST_ ranged from 0.006 to 0.148 and was generally higher (>0.06) between Europe and North America. High *F*_ST_ was observed between isolated populations such as Gilbert Bay, Lake Ogac, and Lake Tariujarusiq and all other samples. IBD was detected across the range (*r* = 0.682, *p* < 0.001) and remained robust to regional evaluation (western Atlantic *r* = 0.527, *p* < 0.001; Greenland/Iceland with the exclusion of the Arctic lakes *r* = 0.494, *p* < 0.001; East Atlantic *r* = 0.693, *p* < 0.001) (Figure 3). Observations between the West Atlantic and other regions, however, exhibited notably higher *F*_ST_ for any given distance than those within the West Atlantic or Greenlandic/Icelandic/East Atlantic systems. Welch *t* test comparison of these two groups revealed significantly divergent *F*_ST_ means (*t *= −62.996, *df *= 953.2, *p* < 0.001).

**Figure 4:**
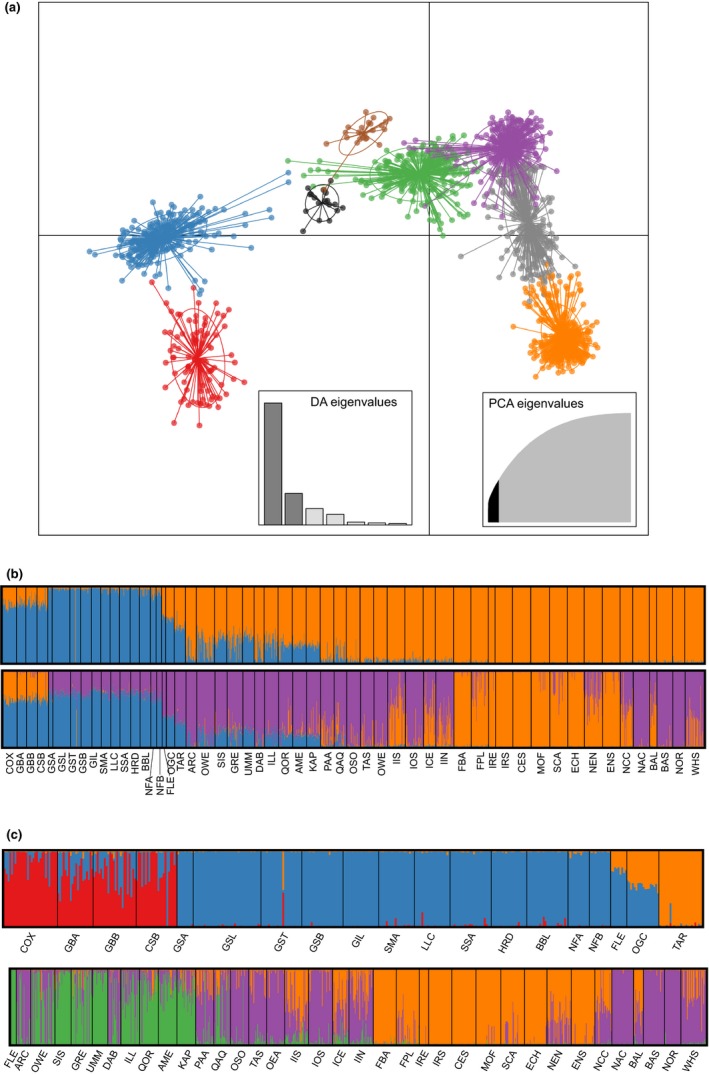
Analyses of population structure. a) Scatter plot of the first and second discriminant functions of DAPC analysis of 8 clusters delineated by the find.clusters function in ADEGENT. Clusters are approximately geographically representative and are color coded as RED = Gulf of Maine, BLUE = Atlantic Canada, GREEN = West Greenland, PURPLE = Offshore Europe, GRAY = northern Europe, ORANGE = southern Europe, BLACK = Ogac, and BROWN = Tariujarusiq. (b) Admixture plots from structure analysis showing all samples at K = 2 (top) and K = 3 (bottom). (c) Hierarchical structure analysis in North America (top) and Europe (bottom). Note that the Flemish Cap (FLE) is included in both hierarchal runs for comparison

**Figure 5:**
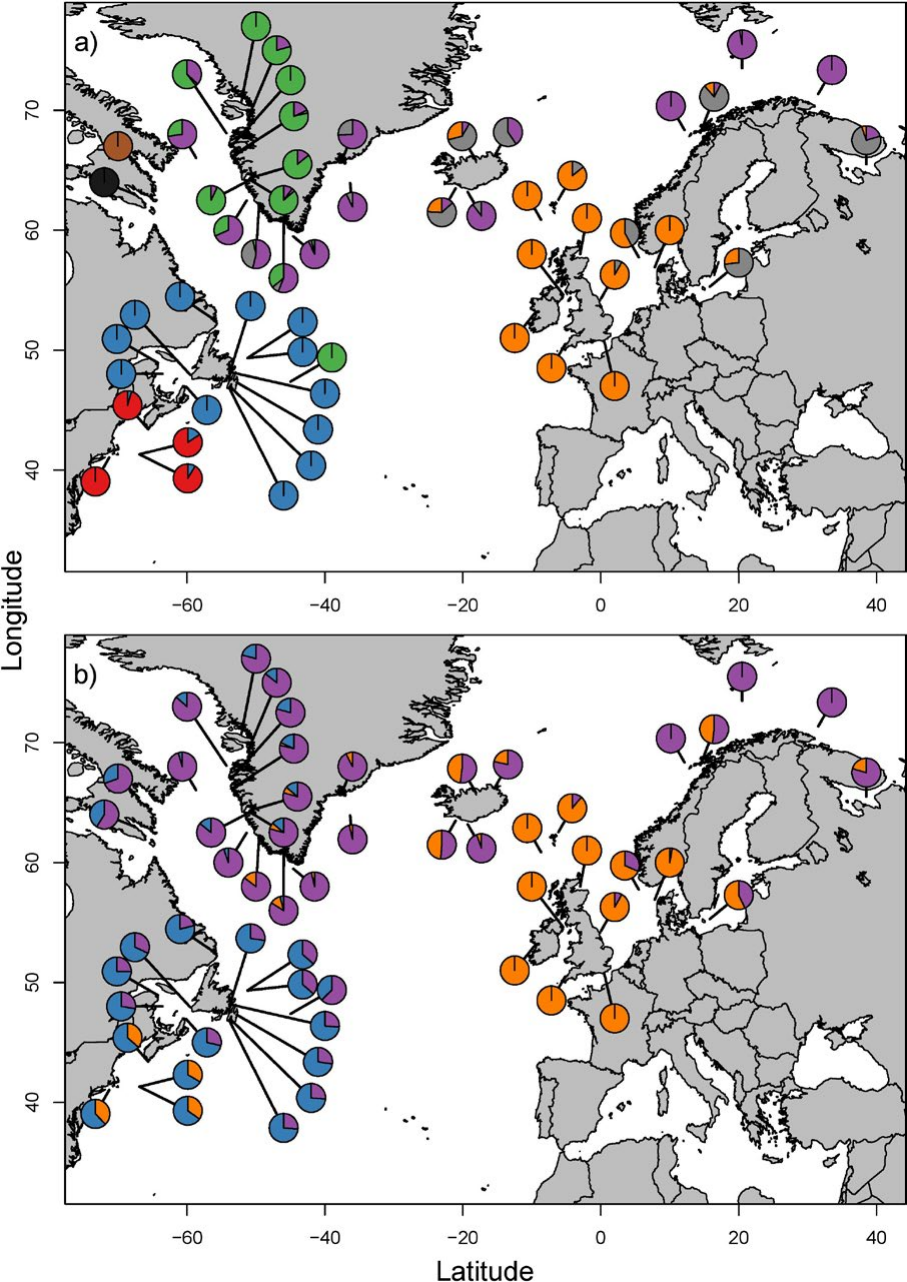
Geographic distribution of clusters from a) DAPC and b) structure (*K* = 3) analyses. DAPC colors are RED = Gulf of Maine, BLUE = Atlantic Canada, GREEN = West Greenland, PURPLE = Offshore Europe, GRAY = northern Europe, ORANGE = southern Europe, BLACK = Ogac, and BROWN = Tariujarusiq

For DAPC, the first 62 PCs were retained, indicated to be the optimal number by the optim.a.score function and representing 49.4% of total variation in the dataset. Eight clusters displayed the most favorable BIC score. The first and most informative discriminate function, which describes 65% of the variance (Figure 4a), distinctly separates clusters in the North America from those in the Europe. The North American clusters approximate the Gulf of Maine and Atlantic Canada, respectively, while three European clusters approximately represent West Greenland, northern Europe (including Iceland), and southern Europe, while a fourth cluster, termed Offshore Europe, is found most commonly among samples among offshore European and Greenlandic samples (Figure 5a). Lake Ogac and Lake Tariujarusiq form their own clusters located intermediate along the first discriminate function to the North American and European clusters, while individuals from the Flemish Cap cluster almost exclusively with West Greenland.

In our structure analyses, the best supported *K* was *K* = 2, which indicated division between the North America and Europe and a clinal introgression of North American genotypes in Greenland (Figure 4b). For *K* = 3, southern Europe is additionally separated from northern Europe and Greenland in a pattern qualitatively similar to the DAPC results, although support for *K* = 3 is significantly lower. Hierarchical structure analysis in the West Atlantic best supported two clusters that were reflective of a North–South divide between the Gulf of Maine and Atlantic Canada (Figure 4c and 5b). A more complex northwest–southeast structure is evident in the East Atlantic, where West Greenland samples separate from Offshore Europe to form a second and third cluster in addition to southern Europe (Figure 4c). There is strong admixture within coastal samples in Norway and Iceland but interestingly offshore samples from these regions share ancestry with West Greenland, as do the Flemish Cap and the Davis Strait.

*F*_ST_ for 96 loci was above the 95% confidence interval, and 44 above the 99% confidence interval following analysis in arlequin. Initially, bayescan detected 424 outlier loci, declining to 181 after controlling for transatlantic structure. Outliers detected by bayescan included the majority of arlequin outliers (75% at *a* = 0.05, 93% at *a* = 0.01). Outliers chosen for further analyses comprised a set of 68 loci common to either of the bayescan runs and arlequin analyses at *a* = 0.05. Population structure with putatively neutral loci displayed a corresponding east–west structuring pattern as with the full panel. The main effect of outlier filtering was a breakdown of North–South population structure on both sides of the Atlantic, which resulted in a reduction from six DAPC clusters to four (excluding the Arctic lakes). These four clusters included a single West Atlantic cluster, a European cluster, and equally strong separation between inshore Greenland and offshore Arctic populations.

### 3.3 Migration

**Figure 6:**
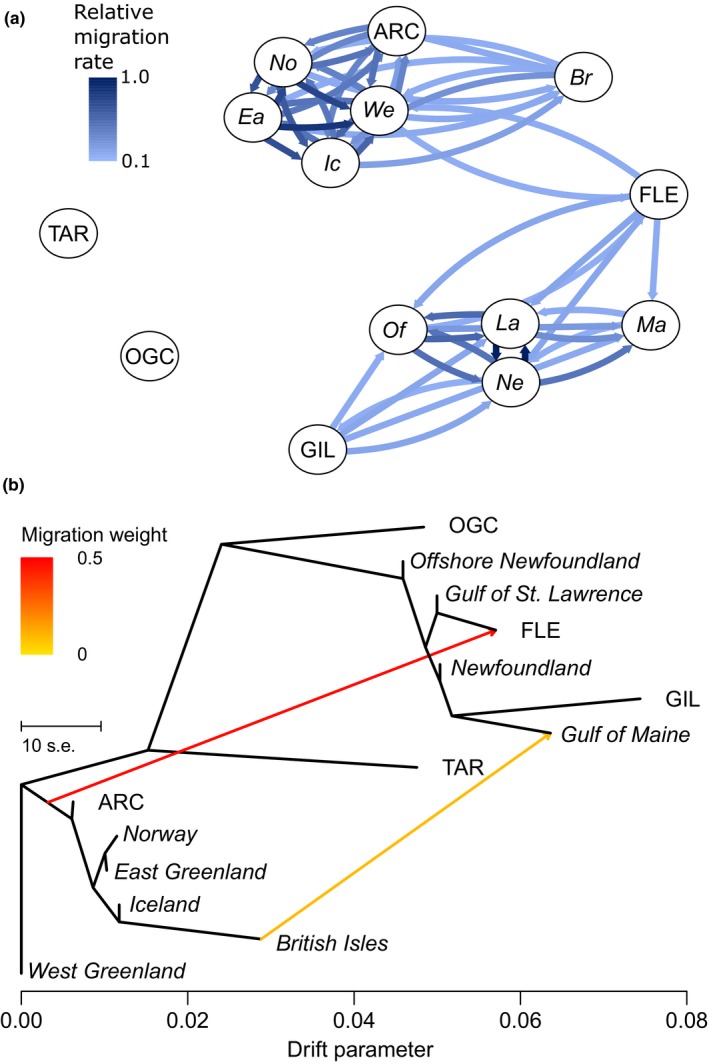
Migration estimated by (a) divMigrate based on GST and (b) treemix based on two migrations. Samples are grouped geographically as follows: Gulf of Maine (Ma) = COX, GBA, GBB, CSB; Gulf of St. Lawrence (La) = GSA, GSL, GST, GSB; Newfoundland (Ne) = SMA, LLC, SSA, HRD, BBL; Offshore Newfoundland (Of) = NFA, NFB; West Greenland (We) = OWE, SIS, GRE, UMM, DAB, ILL, QOR, AME, KAP, PAA, QAQ, OSO; East Greenland (Ea) = TAS, OEA; Iceland (Ic) = IIS, IOS, ICE, IIN; British Isles (Br) = FBA, FPL, IRE, IRS, CES, MOF, SCA, ECH, NEN, ENS, NCC, BAL; Norway (No) = NAC, BAS, NOR, WHS

Analysis of asymmetric migration further supported the presence of North American and European clusters; divMigrate showed high levels of migration between all groups within North America and Europe while treemix splits Europe from North America at the terminal node of the dendrogram (Figure 6). treemix results suggest that the Flemish Cap comprises North American ancestry but are subject to a strong migration event from the Europe (Figure 6b). Further, a relatively strong (>0.1) migration is detected directly between West Greenland and the Flemish Cap by divMigrate, along with continued migration from the Flemish Cap into various West Atlantic populations (Figure 6a). In the best supported treemix model, a second, weaker migration is also detected from southern Europe to the Gulf of Maine (Figure 6b).

## 4 DISCUSSION

The Pleistocene spanned a period of repeated glacial cycles that resulted in the retreat of many North Atlantic marine species into glacial refugia, leaving distinct phylogenetic signatures (Maggs et al., 2008; Provan & Bennett, 2008). During the LGM, one of the Pleistocene's most severe climatic events, Atlantic cod populations are thought to have retreated into refugia in the East and West Atlantic, and a subsequent westward recolonization of Greenland occurred from the East (Bigg et al., 2008). Previous studies using allozyme, microsatellites, and mitochondrial markers have detected pronounced genetic differentiation between East and West Atlantic cod (Carr & Marshall, 2008; Mork et al., 1985; O'Leary et al., 2007; Pogson et al., 1995) that is consistent with the separation of cod populations into glacial refugia during the LGM. In our study, glacial vicariance remains strongly supported by the differentiation between North America and Europe that dominates all population structure analyses. This separation is robust to the removal of outliers, suggesting that while latitudinal structure may be influenced by parallel adaptation, transatlantic structure is largely governed by neutral processes (Bradbury et al, 2010). Where previous studies supporting transatlantic vicariance may have been confounded by the possibility of IBD due to large geographic distances between samples (Pampoulie, Stefánsson, Jörundsdóttir, Danilowicz, & Danielsdottir, 2008; Pogson et al., 1995), our higher resolution of sampling sites suggests greater differentiation between North American and European populations than within those regions, even when shelf sea distance is controlled for. Similar patterns are observed by Lait et al. (2018), who, using analysis of the mitogenome, observe significant differentiation between many European and North American samples and an absence of population structure or IBD within regions. The role of glacial refugia in contemporary population structure is supported by East–West structure in other North Atlantic shelf species. For example, capelin (*Mallotus villosus*), a pelagic species with high larval dispersal, exhibit deep divergence between the West Atlantic and the East and Central Atlantic (Dodson, Tremblay, Colombani, Carscadden, & Lecomte, 2007). Similarly, East and West Atlantic population structure is observable in Atlantic wolffish (*Anarhichas lupus*), although phylogeographic history may be only one of numerous influences (McCusker & Bentzen, 2010).

At the end of the LGM, increased availability of cod habitat is thought to have opened the Greenlandic coast to expansion from Europe (Bigg et al., 2008). Correspondingly, in all our analyses Greenlandic samples show a high affinity with Europe while exhibiting a much lower affinity to North America (Figure 5). In DAPC, in particular, there is a longitudinal gradient between Greenland and Europe, while separation from North America is essentially complete (Figures 4a and 5a). The two DAPC clusters identified in Greenland were previously identified by Therkildsen et al. (2013) using the same data, but our inclusion of a broader dataset places them in context with the rest of the North Atlantic. For example, the East Greenland cluster identified by Therkildsen et al. (2013) (and termed the Offshore cluster herein) extends almost across the breadth of the European range (Figure 5a) and appears to link offshore populations of Northeast Arctic cod, Icelandic Frontal cod, and Davis Strait cod. This homogeneity may imply shared ancestry or ongoing connectivity among offshore populations (see also Bonanomi et al., 2016), which may have significantly influenced westward expansion following the LGM. Colonization of the subarctic may have extended beyond Greenland, leading to secondary contact between European and North American populations.

One of the most remarkable examples of putative secondary contact is the Flemish Cap, a shallow (125–700 m) remote bank off the easternmost tip of the Newfoundland Shelf (Stein, 2007). The link between the Flemish Cap and European populations is evident from Bentzen, Taggart, Ruzzante, and Cook (1996) and Bradbury et al. (2013), who find affiliations with several East Atlantic populations, although purely North American haplotypes are found using the mitochondrial genome (Lait et al., 2018). Using DAPC and structure on a wider range of European data, we find that the Flemish Cap population is dominated by a Greenlandic cluster found at no other North American locations, implying that the Flemish Cap population represents an offshoot of Greenlandic ancestry in North America. Such observations may result from cod eggs and larvae from Greenland that occur in the Davis Strait (Wieland & Hovgard, 2002) being transported south by the Labrador Current and becoming trapped by an oceanic gyre surrounding the Cap (Gil, Sánchez, Cerviño, & Garabana, 2004; Stein, 2007). Subsequently, adults may then be prevented from leaving by the gyre and the 1,000 m deep Flemish Pass that separates the Flemish Cap from the Newfoundland Shelf (Stein, 2007). treemix results find strong signal of migration between the Davis Strait and the Flemish Cap, but also indicates that the Flemish Cap population is largely of North American ancestry (Figure 6b). This is supported by the presence of North American genotypes at the Flemish Cap detected by structure. Other results suggest that migration between Greenland and the Flemish Cap is via some route other than the Davis Strait. For example, the DAPC Offshore cluster is dominant at the Davis Strait but absent from the Flemish Cap (Figure 5a), while divMigrate suggests migration is restricted to that occurring directly between West Greenland and the Flemish Cap. Thus, while our results indicate strong support for Greenlandic origin of the Flemish Cap population, they remain inconclusive as to the exact route or mechanism of migration.

Strong European influence has also previously been found among cod in meromictic lakes on Baffin Island, including Lake Ogac and Lake Tariujarusiq (Bradbury et al., 2013; Carr & Marshall, 2008; Lait et al., 2018). The resident populations occurring in Ogac and Tariujarusiq were established 5 to 8 kya following a period of comparatively warm climate, rising sea levels, and isostatic rebound that enabled cod to inhabit the then fully marine sites (Hardie, Gillett, & Hutchings, 2006). Westward colonization following glacial retreat supports the hypothesis of at least some colonization from Europe (Lait et al., 2018; Therkildsen et al., 2013). However, our results concerning these populations were inconclusive. In DAPC analysis, each lake affiliated to its own cluster, indicating high genetic distinctiveness that is likely the result of small population size and strong genetic drift (Hardie et al., 2006, 2008 ). Although slightly closer to European clusters, these clusters were ultimately intermediate to Europe and North America (Figure 4a). Conversely, at *K* = 2 both lakes exhibit North American affinity in structure analysis, although *K* = 3 introduces some admixture from Greenland. Lait et al. (2018) find evidence of paraphyly in the geographically and ecologically similar Lake Qasigialiminiq, and hypothesize multiple colonization events from both the East and West Atlantic, but note that further study is required for a better understanding of the history of meromictic lake populations.

Our study focuses largely on genome‐wide patterns; however, more recent research emphasizes the roles of genomic architecture, in particular chromosomal inversions on linkage groups (LGs) 01, 02, 07, and 12, in shaping both historical and contemporary population genomic processes. For example, ecotype associated inversions likely play a significant role in different migratory behaviors between Northeast Arctic cod and Norwegian coastal cod (Berg et al., 2016), and are associated with migration behavior in numerous populations (Barney, Munkholm, Walt, & Palumbi, 2017; Berg et al., 2017; Sinclair‐Waters et al., 2017). Although the inversions on both sides of the Atlantic are thought to have common ancestral origin (Berg et al., 2017), the shared ancestry between offshore (migratory) ecotypes implied by our results is contradicted by the findings of Kirubakaran et al. (2016), who find the nonmigratory Norwegian coastal cod to represent the ancestral population (in Norway and for the LG01 inversion at least). This implies that our finding of a common Offshore cluster may be the product of parallel adaptation driven by one or more inversions. Interestingly, despite using a North American SNP panel, we are less able to reproduce migration associated divergence in North America than in Europe (e.g., Barney et al., 2017; Sinclair‐Waters et al., 2017). In some cases, such as the northern cod, this may reflect the finding that population structure at inversion sites seems to operate at the individual level (i.e., populations homozygous for one of the other form of an inversion appear to exist in sympatry (Kess et al., 2018). Other patterns observed in our data, for example, in Baltic Sea and north Sea divergence, may reflect contemporary local adaptation to a salinity and temperature gradient (Berg et al., 2015), and we found that removing putatively adaptive (outlier) loci from our analysis resulted in the North Sea and Baltic Sea clustering together. Adaptation to temperature and salinity has again been linked to chromosomal inversions on LGs 02, 07, and 12 and can lead to adaptive divergence at both large and small geographic scales (Barth et al., 2017; Bradbury et al, 2010; Sinclair‐Waters et al., 2017). Of the outliers we encounter, there is an even distribution among LGs, while in our full dataset each of LGs 01, 02, 07, and 12 is represented by only a small number of loci (11–33). The resultant stochasticity makes it difficult to draw meaningful conclusions regarding the phylogeography of the inversions from our dataset alone. However, chromosomal inversions clearly play a significant role in the phylogeographic history of Atlantic cod and future research will surely continue to explore this role.

The study of phylogeographic histories is relevant to a diversity of conservation and management applications in marine species (e.g., Sinclair‐Waters et al., 2017; von der Heyden, 2017), but studying phylogeographic patterns in species with oceanic ranges is a significant challenge. Here, we address this challenge in Atlantic cod by synthesizing three pre‐existing SNP genotype datasets to improve representation across the species’ range. Our dataset includes 1,494 individuals taken from 54 Atlantic‐wide locations, representing one of the most geographically and genetically extensive datasets for a marine fish to date. (Note, however, that Arnason (2004) achieves greater geography coverage than us, albeit using a much‐reduced genome representation, and that despite lower geographic coverage, modern population genetics routinely employs a larger genomic coverage.) Our approach circumvents the expense of novel data collection and illustrates the potential for collaborative synthesis to address phylogeographic questions (Helyar et al., 2011; Morin et al., 2004). The benefits of a synthesis approach are especially apparent in marine fishes, in which the acquisition of data is both challenging and expensive (Albaina et al., 2013; Bernatchez et al., 2017).

Our final dataset of 796 SNP markers is moderate by contemporary standards and, aside from the limits of the source data, was primarily limited by the degree of marker overlap. The use of the CGP dataset resulted in all common markers in our analysis originating from a North American broodstock, increasing the risk of ascertainment bias affecting our results. We observe a marked decline in heterozygosity from North America to Europe that we attribute to ascertainment bias. In general, however, the impacts of ascertainment bias in high gene flow marine species are thought to be modest (Bradbury et al., 2011). Further, we would therefore expect a bias toward greater diversity represented in North America to favor detection of North American variants in Europe, but instead, we primarily see the reverse (European genotypes in North America), giving us confidence that the bias has a negligible impact on our results. Another potential source of bias was filtering for strand mismatch. In the absence of records that would have allowed us to track the sequenced strand, we mitigated data loss by correcting ambiguous mismatched strands (i.e., A/T and C/G flips, for which alternate strands were confounded with alternate alleles) by comparing minor allele frequencies between samples of the same putative populations between datasets. We found that only variants with a MAF of ≤0.45 could be reliably identified this way, resulting in a slight systematic bias toward reduced heterozygosity when ambiguous markers were included. However, all duplicated analyses yielded nearly identical between the inclusion and exclusion of MAF resolved genotypes, again suggesting that the impact of this bias was negligible.

## 5 CONCLUSIONS

The LGM was a crucial event in the phylogeographic history of North Atlantic marine species, as indicated by the observed strong phylogenetic signals. We investigated these signals in Atlantic cod using a data‐synthesis approach to generate a single range‐wide dataset. In doing so, we reveal new details of the genetic impacts of glacial vicariance, such as secondary contact via the Davis Strait and the Flemish Cap. Further, we demonstrate the utility of a data‐synthesis approach to genetics and ecology. Current “whole genome” studies now often employ millions of markers, while the deployment of hundreds to thousands of SNPs has become more routine in ecology and conservation research. Synthesis of common datasets, as demonstrated here, may be increasingly applicable because of increased accessibility of genomic approaches and archived datasets across target species.

## AUTHOR CONTRIBUTIONS

The study was conceived by GRC in discussion with SJH, JHH, and IRB, with contributions to the design by all authors. GRC and MdB supervised RF's research program. Data were provided by IRB, JHH, NOT, SJH, and PB. Data synthesis and analysis were carried out by RF with assistance from IRB, SJH, and JHH and suggestions from all other authors. The manuscript was drafted by RF and IRB and was read and revised by all authors.

## DATA ACCESSIBILITY

The SNPs analyzed in this study are available in GenBank. Accession numbers are listed in the Supporting information Appendix S2. Genotypes are available on Dryad (https://doi.org/10.5061/dryad.66jp1m9).
